# What distinguishes emotion-label words from emotion-laden words? The characterization of affective meaning from a multi-componential conception of emotions

**DOI:** 10.3389/fpsyg.2024.1308421

**Published:** 2024-01-23

**Authors:** Ángel-Armando Betancourt, Marc Guasch, Pilar Ferré

**Affiliations:** Departament de Psicologia and CRAMC, Universitat Rovira i Virgili, Tarragona, Spain

**Keywords:** emotion-label words, emotion-laden words, component process model, random forest, valence, feeling, interoception

## Abstract

Past research that distinguishes between affective and neutral words has predominantly relied on two-dimensional models of emotion focused on valence and arousal. However, these two dimensions cannot differentiate between emotion-label words (e.g., fear) and emotion-laden words (e.g., death). In the current study, we aimed to determine the unique affective characteristics that differentiate emotion-label, emotion-laden, and neutral words. Therefore, apart from valence and arousal, we considered different affective features of multi-componential models of emotion: action, assessment, expression, feeling, and interoception. The study materials included 800 Spanish words (104 emotion-label words, 340 emotion-laden words, and 356 neutral words). To examine the differences between each word type, we carried out a Principal Component Analysis and a Random Forest Classifier technique. Our results indicate that these words are characterized more precisely when the two-dimensional approach is combined with multi-componential models. Specifically, our analyses revealed that feeling, interoception and valence are key features in accurately differentiating between emotion-label, emotion-laden, and neutral words.

## 1 Introduction

Language contains words that can effectively describe or elicit emotions (i.e., affective words) and words that do not evoke any emotional response (i.e., neutral words). Some researchers argue that affective information plays an important role in how we represent and process words in our minds (Kousta et al., 2011; Citron, 2012). In fact, various studies have demonstrated that affective words have a processing advantage compared to neutral words (hereinafter NT words) (Kousta et al., 2009; Citron, 2012; Vinson et al., 2014). Affective words are not a homogeneous set. We need to distinguish between two types: emotion-label words (hereinafter EM words) and emotion-laden words (hereinafter EL words) (Pavlenko, 2008). EM words explicitly indicate affective states (e.g., joy, anger). In contrast, EL words may elicit an emotion but do not express an emotion directly (e.g., murderer, birthday).

The affective content of words is usually characterized in terms of valence and arousal. Valence refers to the extent to which a stimulus is pleasant or unpleasant (e.g., “fear” is an unpleasant/negative EM word and “murder” is an unpleasant/negative EL word, whereas “joy” is a pleasant/positive EM word and “mother” is a pleasant/positive EL word). Arousal is related to the physiological state and refers to the level of activation (excitement/calmness) provoked by a stimulus (e.g., “tense” is a highly arousing EM word and “war” is a highly arousing EL word, while “relax” is a low arousing EM word and “bed” is a low arousing EL word). These two dimensions (often referred to as “core affect”) are central to the understanding of human emotions (Barrett and Russell, 1999; Russell, 2003). Affective words (EM and EL) differ from NT words in both dimensions. Considering valence, affective words are perceived as highly pleasant or unpleasant, while NT words are neither positive nor negative. In terms of arousal, affective words are perceived as more arousing than NT words; however, the degree of arousal they elicit can vary.

The effects of valence and arousal during word processing have been widely demonstrated, both with behavioral (reaction times, RT) and electrophysiological (event-related potentials, ERPs) measures (see Hinojosa et al., 2020a for a review); however, the findings across different studies have not always been consistent. Studies focused on the effects of arousal during lexical decision and naming tasks have reported mixed results. For instance, Recio et al. (2014) observed slower RTs for low-arousing words, while other studies have found no arousal effects (e.g., Yao et al., 2016). Several studies on valence have reported a faster RT for positive words compared to negative words and NT words (Estes and Adelman, 2008; Hofmann et al., 2009; Kousta et al., 2009; Kuperman et al., 2014; Vinson et al., 2014; Yap and Seow, 2014). However, the effect of negative valence is unclear. Some studies have shown that negative words have a disadvantage in processing (Larsen et al., 2008), others have observed a facilitation (Kousta et al., 2009; Vinson et al., 2014), while others have reported no effect (Larsen et al., 2006; Scott et al., 2014).

The above-mentioned inconsistencies may be partly due to the characteristics of the experimental stimuli. For instance, previous studies have mixed EM and EL words in their experimental lists (Kissler et al., 2009; Kousta et al., 2011; Palazova et al., 2011; Chen et al., 2015). Nonetheless, there is behavioral and neurolinguistic evidence of the differences in processing between these two types of words (Altarriba and Basnight-Brown, 2011; Knickerbocker and Altarriba, 2013; Kazanas and Altarriba, 2016a; see Wu and Zhang, 2020, for a review). The distinction between EM and EL words has been studied in paradigms and tasks such as the affective Simon task (Altarriba and Basnight-Brown, 2011), rapid serial visual presentation (RSVP) paradigm (Knickerbocker and Altarriba, 2013), masked and unmasked priming paradigm (Kazanas and Altarriba, 2015), and lexical decision tasks (Kazanas and Altarriba, 2016b; Martin and Altarriba, 2017). These studies suggest that EM and EL words have distinct patterns of processing. For instance, it has been found that EM words yield faster RTs than EL word (Kazanas and Altarriba, 2016b). In addition, ERP studies have also shown significant differences between EM and EL words. For example, Zhang et al. (2017) found that EM words and EL words evoke distinct cortical responses at different stages of word processing. Their study found that the amplitude of the N170, a component which is sensitive to the distinction between affective and non-affective information, was larger for EM words than for EL words. This result indicates that EM and EL words are differently processed at early stages of processing. However, the analysis of the LPC, a component which is sensitive to the positivity or negativity of a word, showed that negative EM words elicited a larger response in the right hemisphere when compared to positive EM words and to EL words. These findings imply that the neural correlates and hemispheric processing of EM and EL words are different.

Similarly, a more recent study compared EM and EL words in an affective priming paradigm (Wu et al., 2021). This paradigm makes it possible to examine how the presentation of a (prime) word affects the processing of a (target) word presented immediately after. The typical result is a facilitative effect in congruent trials, where both the prime and the target share the same affective polarity (e.g., a prime word with a positive valence and a target word with a positive valence), compared to incongruent trials, in which the prime and target have different affective polarities (e.g., a prime word with positive valence and a target word with negative valence; Klauer, 1997). In the study conducted by Wu et al. (2021), all the targets were EL words, while the primes could be either EM or EL words. A main finding of this study was that EL targets were processed more accurately when they were primed by EL words rather than by EM words.

The study of Wu et al. (2021) shows that, despite the affective congruency/incongruency between the prime and the target, the type of word (i.e., EM vs. EL) also determines affective priming. This suggests that there may be differences in affective content between these two types of words. As mentioned earlier, EM words have inherent affective properties because they refer directly to an affective state. In contrast, the affective content of EL words probably comes from their association with personal experiences (Wu and Zhang, 2020). Betancourt et al. (2023) obtained some evidence of this. These authors examined the associative structure of EM, EL, and NT words using a word association task. In this task, participants are asked to quickly respond to a cue word with the first word that comes to their mind (i.e., an associated word). The authors found that EM cues produced a higher number of EM associates in comparison to EL cues. Importantly, EL cues produced a greater amount of EM associates than NT cues. These results suggest that EM words are strongly connected in the mental lexicon and that the affective content of EL words is acquired by association to affective states.

As previously mentioned, affective content has generally been studied in terms of valence and arousal (e.g., Bradley and Lang, 1999; Barrett and Russell, 1999; Russell, 2003). However, these two variables are not sufficient to differentiate between EM and EL words. On the one hand, both EM and EL words are either positive or negative and tend to be more arousing than neutral words. On the other, the literature reviewed shows that, despite being matched in terms of valence and arousal, EM and EL words behave differently in several experimental paradigms. Therefore, further analysis is needed for an accurate distinction between these two types of words. In this study, we aim to describe the affective content of EM and EL words by examining a set of features, other than valence and arousal, that are related to the emotional experience.

Some theorists suggest that the emotional experience is shaped by multiple factors. Of interest here is the Component Process Model (CPM) of emotion proposed by Scherer and co-workers (Scherer, 2001; Scherer, 2009; Sander et al., 2018), which describes emotions as a complex and dynamic process that involves different response mechanisms. The model identifies five components: (1) cognitive appraisal (*assessment*), (2) physiological activation (*interoception*), (3) motor expression (*expression*), (4) action tendencies (*action*), and (5) subjective feeling (*feeling*; Scherer, 2009; Sander et al., 2018). The cognitive appraisal (hereinafter *assessment*) component involves evaluating the importance of a stimulus by considering its impact on the individual’s wellbeing and survival. The physiological activation (hereinafter *interoception*) refers to detecting internal bodily changes like increased heart rate, muscle tension, or sweating. The motor expression (hereinafter *expression*) component encompasses various forms of expression, such as facial expressions, vocal expressions, body movements, gestures, and posture. The action tendencies (hereinafter *action*) component refers to a readiness to act in a certain way, related to the urge to approach or avoid something to achieve a specific goal. The subjective feeling (hereinafter *feeling*) component is shaped by an integrated awareness of the previous components, and this integration may result in anger, sadness, or other feelings that can be categorized or verbally labeled according to the semantic profile of emotion words (Scherer, 2009).

Several studies have examined how the components described by the CPM can be helpful in the characterization of EM words (Fontaine et al., 2007; Gillioz et al., 2016; Gentsch et al., 2018; Scherer and Fontaine, 2019). For example, Fontaine et al. (2007) explored the dimensional space that best accounts for the similarities and differences within EM words. Using a principal components analysis, they obtained a four-dimensional solution which included these dimensions: evaluation-pleasantness, potency-control, activation-arousal, and unpredictability. These findings were replicated in three different languages (English, French and Dutch). In a further study, Scherer and Fontaine (2019) conducted a larger-scale analysis using 142 emotion features, finding that the semantic structure of emotion words is consistent with the CPM. A similar approach was adopted by Ferré et al. (2023), who collected subjective ratings for a large set of potential EM words in relation to a series of variables associated with the different components of emotion: action, assessment, expression, feeling, and interoception. They also considered other variables, such as valence and arousal. Feeling and interoception were identified as the most relevant predictors of emotion prototypicality (i.e., the extent to which a word exemplifies an emotion). That is, words were more likely to be considered as good exemplars of the “emotion” category if they were associated greatly with feelings and internal bodily sensations (interoception). This result indicates that these two variables are crucial for defining the emotional experience.

The above-mentioned studies, which focused on EM words, highlight the importance of incorporating the variables proposed by the CPM into research on the affective content of words. The aim of this study was to examine whether the most relevant affective characteristics of EM words are also useful to differentiate EM words from EL words, and if they distinguish these two types of words from neutral words. We used the same framework as Ferré et al. (2023) and examined the role of a set of variables related to the different components of the emotional response, as well as the role of valence and arousal. We collected ratings for a large set of EM, EL, and NT words in relation to assessment, interoception, expression, action, and feeling. We used a double approach with these ratings. First, we created a semantic space using a Principal Component Analysis (PCA) to provide information about the organizational structure of EM, EL and NT words. Second, we made a prediction model using a Random Forest Classifier to identify the variables that most predicted whether a word belonged to a certain type. Based on the findings of Ferré et al. (2023), we expected feeling and interception to be the most important predictors of word membership in the EM category. Furthermore, these variables might not have a significant role in the definition of EL words, considering that they do not denote emotions, and thus contribute to the differentiation between EM and EL words.

## 2 Method

### 2.1 Participants

Word ratings were obtained from 386 participants. The final number of valid participants after data cleaning (see below) was 370 (318 females, 85.95%, and 52 males, 14.05%), whose mean age was 19.46 (SD = 3.64). Participants were students at the Universitat Rovira i Virgili in Tarragona, Spain. Each participant completed one or more questionnaires in exchange for academic credits or as a volunteer. All participants signed an informed consent form before starting the ratings. The research was conducted in line with the APA ethical standards. Approval was granted by the Ethics Committee for Research on People, Society and the Environment of the Universitat Rovira i Virgili (CEIPSA-2021-PR-0044).

### 2.2 Materials

The materials for this study included 800 Spanish words from different sources. A total of 104 Spanish EM words were obtained from the Pérez-Sánchez et al. (2021) database, which contains 1,286 words rated in emotional prototypicality, that is, the degree to which a word refers to a human emotion. The selected words included nouns with a prototypicality score greater than or equal to 3 (in a scale from 1 = “this word does not refer to an emotion,” to 5 = “this word clearly refers to an emotion”), and with a frequency per million score (taken from Duchon et al., 2013) of 1 or higher (see a similar criteria in Gillioz et al., 2016). The selected EM words had an average prototypicality rating of 3.73 (*SD* = 0.51). We discarded derivatives of the same word (e.g., *ilusión* [excitement] vs. *desilusión* [disappointment]; discarded word) and words with different or ambiguous meanings (e.g., *éxtasis* [ecstasy]).

The 696 additional words (EL and NT words) were taken from Stadthagen-Gonzalez et al. (2017), a database that contains 14,031 Spanish words that are rated in valence and arousal. In order to select EL and NT words, and not to include EM words by mistake, we crossed this database with that of Pérez-Sánchez et al. (2021), which only contains EM words. We removed the words in common between both datasets. This left us with only EL and NT words. Then we only included EL and NT words that had a frequency per million greater than or equal to 1 (taken from Duchon et al., 2013). We randomly selected 696 words from this pool and checked them to be sure that no word explicitly labeled an emotion. We used valence values to classify these words into EL and NT. EL words had a valence rating < 4 or > 6, indicating a negative and a positive valence, respectively. Neutral words had a valence rating ≥ 4 and ≤ 6 (Stadthagen-Gonzalez et al., 2017). The final selection included 104 EM words, 340 EL words, and 356 NT words.

### 2.3 Procedure

We focused on seven dimensions: valence, arousal, action, assessment, expression, feeling, and interoception. The ratings for valence and arousal were taken from Stadthagen-Gonzalez et al. (2017). The ratings for the CPM variables (action, assessment, expression, feeling, and interoception) were obtained through a series of questionnaires which were created and administered online using TestMaker (Haro, 2012). The questionnaires for each variable were divided into five versions. Each version contained the same set of randomly assigned words for the five variables. We ended up with 25 questionnaires with 160 words per questionnaire (eight pages each with 20 words per page). The order of presentation of the words was randomized for each participant. None of the participants who completed more than one questionnaire repeated the same set of words and variables. Participants were instructed to rate each word using a 1-to-7 scale (see Table 1 for instructions). Each questionnaire had an option to indicate that the participant was not familiar with a given word (“*No conozco la palabra,”* I do not know the word).

**Table 1:** Instructions.

Variable	Instruction	Scale (from-to)
Action	I relate this word to taking action, doing something, and influencing	1 “strongly disagree” to 7 “completely agree”
Assessment	I relate this word to situations that are important for my wellbeing and/or my survival	1 “strongly disagree” to 7 “completely agree”
Expression	I relate this word to alterations/changes in my body	1 “strongly disagree” to 7 “completely agree”
Feeling	I relate this word to feelings	1 “strongly disagree” to 7 “completely agree”
Interoception	I relate this word to internal bodily sensations	1 “strongly disagree” to 7 “completely agree”
Valence	I consider this word to be highly/slightly unpleasant or highly/slightly pleasant	1 “unpleasant” to 9 “pleasant”
Arousal	I consider this word to be highly/slightly calming or highly/slightly exciting	1 “calming” to 9 “exciting”

Valence and arousal ratings were taken from Stadthagen-Gonzalez et al. (2017).

The dataset used in the present study can be found in online repositories in an Open Science Framework (OSF) repository at https://osf.io/hxcm2/?view_only=74adb248fc88443fabc5ad1daa6abbc6.

## 3 Results

### 3.1 Data cleaning and descriptive statistics

Sixteen participants were eliminated from the total pool of 386. The criteria to eliminate a participant were: (1) A high percentage of identical ratings (i.e., they rate more than 95% of the words in a questionnaire with the same score), and (2) A low correlation between the participant ratings and those of the group who filled out the same questionnaire (correlation lower than 0.1). The final number of valid participants was 370 (mean = 37.04 participants per questionnaire: min = 30, max = 47, *SD* = 4.75). The descriptive values for each variable, and for each word type are shown in Table 2.

**Table 2:** Characteristics of EM, EL, and NT words.

	EM	EL	NT
Action	5.04 (±0.61)	3.98 (±0.98)	3.27 (±0.93)
Assessment	4.56 (±1.15)	4.07 (±1.09)	3.37 (±0.76)
Expression	5.23 (±0.55)	3.71 (±0.87)	2.77 (±0.71)
Feeling	5.80 (±0.58)	3.65 (±1.04)	2.54 (±0.76)
Interoception	5.37 (±0.63)	3.64 (±0.89)	2.67 (±0.69)
Valence	4.61 (±2.49)	5.85 (±1.81)	5.28 (±0.50)
Arousal	6.26 (±1.47)	5.39 (±1.24)	5.07 (±0.72)

All values are means ± SD.

### 3.2 Reliability and validity

We assessed the interrater reliability of the measures using a split-half procedure and computing the Spearman-Brown coefficient with the participants’ ratings. The average Spearman-Brown coefficient was *r* = 0.94 for action (ranging from 0.94 to 0.96), *r* = 0.94 for assessment (ranging from 0.92 to 0.95), *r* = 0.95 for expression (ranging from 0.93 to 0.95), *r* = 0.95 for interoception (ranging from 0.93 to 0.96), and *r* = 0.97 for feeling (ranging from 0.96 to 0.98).

We also examined the validity of our ratings by comparing the ratings collected in the questionaries with those reported in previous normative studies. This was based on the 103 words that overlapped with the study of Ferré et al. (2023). We found moderate to high Pearson correlations for all the variables: action: *r*(101) = 0.49, *p* < 0.01; assessment: *r*(101) = 0.92, *p* < 0.01; expression: *r*(101) = 0.55, *p* < 0.01; feeling: *r*(101) = 0.54, *p* < 0.01; and interoception: *r*(101) = 0.40, *p* < 0.01.

### 3.3 PCA analysis, feature distribution and semantic space

Principal Component Analysis (PCA) is a statistical procedure that helps to reduce dimensionality (i.e., the total number of features in a dataset) while retaining the highest amount of information (Jolliffe and Cadima, 2016). Dimensionality is reduced by transforming the data into a new set of variables called principal components. The assembly of each component is typically based on a correlation matrix which measures the relationship between each feature within the dataset. PCA helps to determine whether samples can be grouped by assessing similarities and differences between them. Observations are generally represented using a coordinate system that makes it possible to identify each observation in a two-dimensional space (Jolliffe, 2002). We reduced dimensionality using a PCA with varimax rotation and Kaiser normalization using SPSS (version 29) and XLSTAT (Addinsoft, 2023). A correlation matrix (see Table 3) was used as the input format for the PCA. Our data obtained a Kaiser-Mayer-Olkin (measure of sampling adequacy) index value of 0.829, which indicates that the correlation matrix is adequate for the analysis. Components with eigenvalues below 1.0 or which accounted for less than 10% of the variance were not considered when the number of components was selected. We ended up with a solution containing two principal components (see Table 4). Principal Component 1 (PC1) explained a total variance of 59.69%, while Principal Component 2 explained a total variance of 25.04%, with a cumulative proportion of explained variance of 84.73% after varimax rotation.

**Table 3:** Correlation matrix (Pearson coefficients).

Variables	Interoception	Expression	Assessment	Feeling	Action	Valence	Arousal
Interoception		**0.90**	**0.68**	**0.89**	**0.77**	0.06	**0.33**
Expression	**0.90**		**0.64**	**0.89**	**0.78**	−0.01	**0.40**
Assessment	**0.68**	**0.64**		**0.59**	**0.67**	**0.51**	−0.06
Feeling	**0.89**	**0.89**	**0.59**		**0.78**	−0.02	**0.35**
Action	**0.77**	**0.78**	**0.67**	**0.78**		**0.07**	**0.35**
Valence	0.06	−0.01	**0.51**	−0.02	**0.07**		**−0.53**
Arousal	**0.33**	**0.40**	−0.06	**0.35**	**0.36**	**−0.53**	

Values displayed in bold are significant at an alpha level of 0.05.

**Table 4:** Variable loadings after Varimax rotation.

Variables	PC1	PC2
Interoception	**0.945**	
Expression	**0.941**	
Assessment	**0.780**	0.496
Feeling	**0.925**	−0.137
Action	**0.892**	
Valence	0.127	**0.910**
Arousal	0.350	**−0.799**

Loadings smaller than 0.10 are not shown. Variables were included in a component if they had values equal to or greater than 0.5. Values in bold indicate the variables which belong to each component.

Table 4 shows the outcomes of performing a PCA across all the variables with the varimax rotation. PC1, which explains most of the model variance (59.69%), is formed by interoception, expression, assessment, feeling, and action. PC2, which accounts for 25.04% of the total variability, is formed by valence and arousal. Furthermore, although assessment did not constitute a component of PC2, it still had a considerable load in that factor.

**Figure 1:**
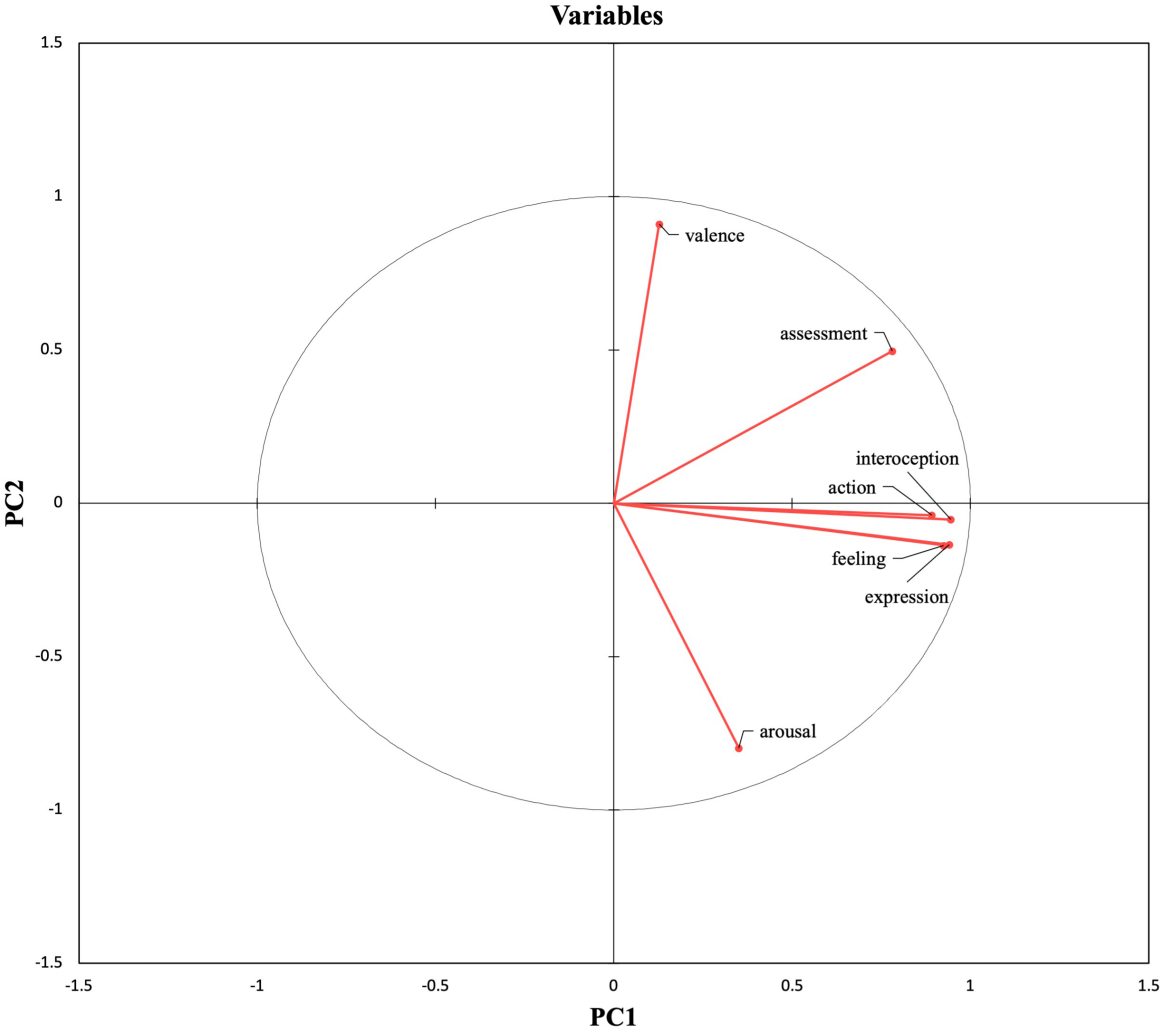
Feature projection.

PC1 accounts for most of the variability in the dataset, and it is mainly constructed with the CPM variables. The variables within PC1 exhibited positive loadings, indicating that all of them are positively correlated. In other words, the features that make up PC1 share a common underlying component that causes them to increase or decrease together. The positive correlation between these features can be observed in Figure 1, in which they are plotted on the right side of the figure.

PC2 accounts for a smaller amount of variance. PC2 has a positive loading for valence and a negative loading for arousal, which means that the variables in this component tend to move in opposite directions. In fact, when we analyzed the correlation coefficients between the features within each Principal Component, valence and arousal exhibited a negative correlation (−0.525). The sign difference in PC2 loadings is visible in Figure 1, in which valence is projected at the top of the figure while arousal is projected at the bottom.

**Figure 2:**
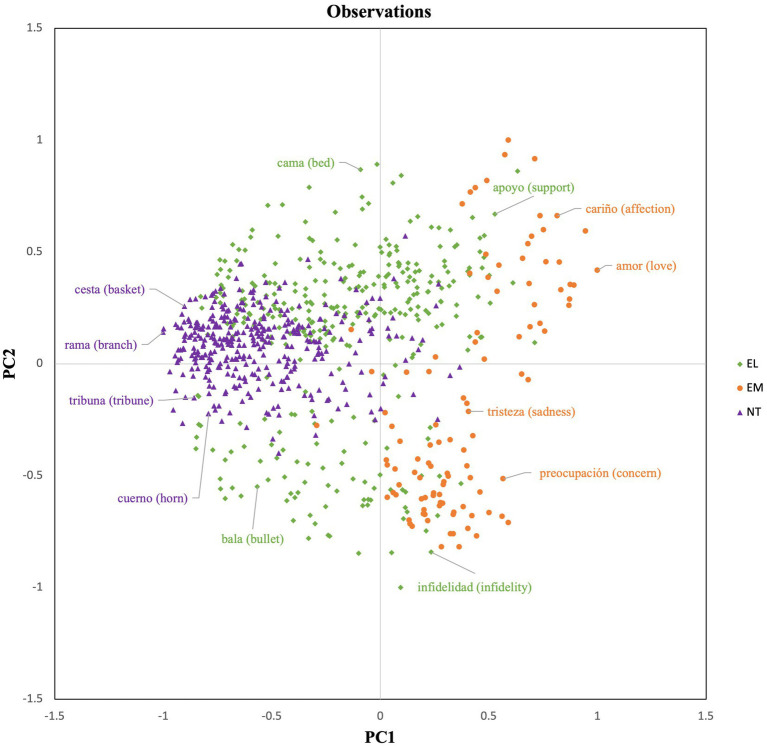
Word projection.

We used the component scores after varimax rotation as a coordinate system to represent the distribution of each word in a two-dimensional space (see Figure 2). As shown in Figure 2, NT words are primarily projected to the left-center side of the figure, while EM words tend to be projected to the right upper and lower side. On the contrary, EL words are distributed across the entire figure with a tendency to be on the upper and lower sides. Figure 2 suggests that NT words tend to show a low score for the CPM variables and show average valence and arousal values. EM and EL words have a similar relationship with PC2, by exhibiting a polarized projection towards the upper or lower part of the figure. However, there is a clear distributional distinction in terms of PC1. As Figure 2 shows, the distribution of EM words (e.g., love, sadness) exhibits a closer proximity to the PC1 variables with respect to both EL and NT words, meaning that EM words tend to show a high score for the CPM variables. At the same time, EL words are plotted more closely to the PC1 variables than NT words, which indicates that EL words tend to have a higher score for the CPM variables than NT words.

### 3.4 Random forest classifier

PCA is a useful dimension reduction tool that provides information about the distribution of EM, EL and NT words in a coordinate system in relation to various features. However, it does not directly capture each individual variable’s contribution to the characterization of a particular class of words. The Random Forest Classifier is a useful technique to address this issue. It enables us to determine the impact of each feature on the prediction of whether each particular word belongs to the EM, EL, or NT types (see the Appendix for a detailed explanation of this method).

We included the 800 words of the study in the analysis. Using Python version 3.7.2 and Scikit-learn library (Pedregosa et al., 2011), we created a prediction model using Random Forest Classifier (RFC) with Recursive Feature Elimination (RFE). The model used a total of 10,000 decision trees, had a maximum depth of 7, and a maximum number of features equal to the square root of the total number of features (√7). In addition, we adopted a train-test-split ratio of 75% for training (the portion of the dataset that is employed to train the model) and 25% for testing (the portion of the dataset that is used to test the prediction of the trained model). Before splitting the data into the training and the test datasets, we established a “*random state*,” which controls the randomization of the data so it is reproducible. We created a code that iterates over 200 possible random states to later use the average predictive accuracy of the 200 models. The results showed that the lowest predictive accuracy was 91.5%, the maximum was 98.5%, and the average accuracy of the 200 models was 95.7%. Within the 200 models, no random state reproduced an accuracy of precisely 95.7%. Therefore, we selected the nearest highest model, which had an accuracy of 96%. Therefore, after training the RFC, our final selected model predicted the classes of the unseen dataset (testing data) with 96% accuracy.

We conducted an in-depth analysis to determine the most relevant features for predicting each word class independently (EM, EL, and NT). Feature importance was calculated using the prediction accuracy. We used the Mean Decrease in Accuracy (MDA) for this analysis. MDA is an approach that calculates the increase in error resulting from the performance of the model with and without a feature (Petkovic et al., 2018). This procedure is carried out for all decision trees and features, providing an estimate of the effect of each feature on the accuracy of class prediction. In the context of MDA, a positive contribution from a feature indicates that including it enhances the prediction accuracy for that class. Conversely, a negative contribution implies that a particular variable does not provide additional information, thus decreasing the overall accuracy. The results are shown in Table 5. All the features positively contributed to predicting EM words. The feature that contributed the most was feeling, while action and valence contributed the least. The feature that contributed the most to predicting EL words was valence, while feelings, interoception, and arousal made negative contributions. That is, excluding feelings, interoception, and arousal increases the predictive accuracy for EL words. Finally, valence was the feature that contributed most to predicting NT words. In contrast, the remaining features made negative contributions, indicating that adding these features reduces the model’s ability to correctly identify NT words.

**Table 5:** Mean decrease in accuracy per class.

	EM	EL	NT
Action	9.79%	0.55%	**4.0%**
Assessment	16.69%	0.55%	**4.0%**
Arousal	13.24%	**0.59%**	**4.0%**
Expression	16.69%	1.68%	**4.0%**
Feeling	23.59%	**0.59%**	**2.80%**
Interoception	16.69%	**1.73%**	**4.0%**
Valence	9.79%	28.95%	12.87%

Each value refers to the impact on accuracy of each individual variable. Values in bold negatively impact the class prediction.

## 4 Discussion

Numerous studies on differentiating between affective and neutral words have focused exclusively on a two-dimensional model that relies on valence and arousal. These two dimensions cannot explain the differences between EM and EL words. The main objective of the present study was to identify the distinctive affective features of EM, EL, and NT words. We collected word ratings of various variables related to the different components of the emotional response, according to multi-componential conceptions of emotion (the Component Process Model, CPM). These variables are action, assessment, expression, feeling, and interoception. Then, we carried out a Principal Component Analysis (PCA) and a Random Forest Classifier (RFC) technique based on the ratings of the words in these variables as well as in valence and arousal. The results showed that feeling, interoception and valence are key features for accurately differentiating between EM, EL and NT words.

The PCA yielded a two-dimensional solution with two principal components. PC2 accounts for the least amount of variability and is composed of valence and arousal, with valence being the feature that contributes the most to the explained variability. This factor distinguishes affective (EM and EL) words from neutral words: NT words are characterized by mid-value scores in valence and arousal, while EM and EL words display more extreme values. This result indicates that EM and EL words are associated with a positive or negative emotion and with varying levels of activation, while NT words are not associated with a positive or negative emotion, and they do not elicit strong levels of activation. The relevance of these variables in the clustering of affective and neutral words is in line with two-dimensional models (Barrett and Russell, 1999; Russell, 2003), which have been the most popular for characterizing the affective properties of words as well as studying their influence on linguistic processing (e.g., Kuperman et al., 2014; see Hinojosa et al., 2020b, for a review).

The results of the PCA also show that more than two features are needed to account for the distribution of EM, EL, and NT words in a semantic space. Indeed, although affective and NT words can be distinguished in terms of valence and arousal, these two dimensions are not sufficient to distinguish between EM and EL words, as indicated by the relevance of the other component identified in the analysis. Principal Component 1 (PC 1) accounts for most of the variability and is characterized by the Component Process Model (CPM) variables: action, assessment, expression, feeling, and interoception (Scherer, 2001). Our results show that EM words are more closely related to CPM variables than EL and NT words. In contrast, NT words display low ratings for the CPM variables, while EL words show more variability (from low to high scores, see Figures 1, 2). In fact, EL words are plotted in the space between NT and EM words. This finding aligns with those reported in Betancourt et al. (2023), who found that EL words produced a higher number of EM associates compared to NT words during a free association task, and therefore are more connected to emotional states than NT words.

Moreover, our findings suggest that the speakers perceive EM words as clearly related to an appraisal process that results in a certain assessment, a set of physiological changes, an expressive response, a tendency to act, and a feeling associated with a particular emotion. This highlights the multidimensionality of the states described by EM words and points towards the need to adopt an appraisal-driven componential approach to correctly characterize how we represent EM and EL words in our minds. Previous studies have distinguished between EM and EL words in terms of processing (Knickerbocker and Altarriba, 2013; Kazanas and Altarriba, 2015; Wang et al., 2019; Wu et al., 2021); however only a few studies have been interested in their semantic representation. This work shows, for the first time, that EM and EL words have distinct representational features related to multi-componential affective responses.

This study followed the approach used by Ferré et al. (2023), who aimed to identify the features that define EM words. The authors examined the contribution of CPM variables to the emotion prototypicality of a set of potential EM words, and identified interoception and feeling as the best predictors of emotion prototypicality. This suggests that these variables are closely linked to the affective experience. The results of the PCA are in the same line. We also found that interoception and feeling are among the variables that most contribute to PC1. In particular, interoception was the variable with the highest load in this factor. Therefore, among the CPM variables, interoception and feeling are not only the best variables for characterizing EM words (Ferré et al., 2023), but also the ones that contribute most to distinguishing between EM and EL words.

In addition to describing the semantic space of EM, EL and NT words, we were also interested in the contribution that each individual feature makes to predicting each word type. To this end, we conducted an RFC and analyzed the mean decrease in accuracy (MDA). The results of this analysis indicated that EM, EL, and NT words have unique affective features. Indeed, the characteristic that mainly defines NT words is their valence. This result is not unexpected because, by definition, NT words have valence values between 4 and 6 on a scale that goes from 1 to 9 (Stadthagen-Gonzalez et al., 2017). In other words, affective words are characterized by extreme valence values, while NT words are restrained to mid-range valence values. This suggests that NT words are primarily defined by the absence of a negative or positive (pleasant/unpleasant) emotion. This finding is coherent with the results of the PCA analysis in which NT words were clearly differentiated from affective words in PC2 in terms of valence more than in terms of arousal.

The RFC results revealed that four out of the seven features examined in this study positively influenced the prediction of EL words. These features are valence, expression, action, and assessment. The MDA indicated that valence is the most important predictor of EL words. That is, the defining characteristic of EL words is their positive/negative polarity, more than their arousal. This finding is consistent with the PCA, in which we observed that valence strongly influences the distribution of EL words in the semantic space. In fact, the RFC indicates that only valence stands out as a relevant variable in predicting the three word types (EM, EL and NT words). This finding aligns with research revealing that valence is one of the most important organizing features of words in the mental lexicon (Van Rensbergen et al., 2015; Buades-sitjar et al., 2021; Betancourt et al., 2023).

Apart from valence, other features such as expression, action, and assessment also emerged as influential predictors of words belonging to the EL class, although to a lesser extent. Consequently, it seems that EL words may also activate some bodily and behavioral responses by prompting individuals to evaluate and interpret the significance of a situation concerning different outcomes. However, CPM variables clearly play a greater role in predicting whether words belong to the EM class. In fact, all the affective variables considered in this study (action, arousal, assessment, expression, feeling, interoception, and valence) positively impact the prediction of EM words, and the most important variable is feeling. This is in line with the results obtained in the PCA, showing that CPM variables are determining factors in the distribution of EM words in the semantic space. This result is also in accordance with Ferré et al. (2023), who identified feeling and interoception as the best predictors of emotional prototypicality in EM words. Therefore, both the present results and those from Ferré et al. (2023) highlight feelings and internal bodily sensations as the most distinguishing features of EM words. It is noteworthy that the relevance of the last factor has been evidenced by several studies, which report that interoceptive and somatosensory processes play a major role in generating the emotional experience (e.g., Kreibig, 2010; Nummenmaa et al., 2014). Therefore, the present results suggest that the semantic content of EM and EL words is related to distinct affective features. This may contribute to the differences in emotional processing observed between these two types of words (e.g., Wang et al., 2019; Wu and Zhang, 2019; Zhang et al., 2019, 2020).

A limitation of the current study is the gender imbalance of the sample, with 86% of female participants. This is a common shortcoming of affective (e.g., Soares et al., 2012; Montefinese et al., 2014; Stadthagen-Gonzalez et al., 2017) and non-affective (e.g., Brysbaert et al., 2014; Hinojosa et al., 2021) rating studies. However, cross-gender correlations tend to be very high, indicating a high consistency between women’s and men’s affective ratings (e.g., Redondo et al., 2007; Soares et al., 2012; Montefinese et al., 2014). Despite this, future research should include a more balanced distribution to examine in depth the possible differences between genders and increase the generalizability and ecological validity of the findings. On the other hand, future studies may be conducted to investigate the role of other, non-affective variables, on the distinction between EM and EL words. Both age of acquisition (Pérez-Sánchez et al., 2021; Wu, 2023) and sensory experience (Wu, 2023) are worth to be considered, because they are related with the emotional prototypicality of EM words.

To sum up, the present study confirms that valence is a crucial variable in the organization of the mental lexicon, as it distinguishes affective from neutral words. It also shows that other variables related to the multi-componential experience of emotion need to be considered to differentiate EM and EL words. Among these, feeling and interoception seem to be the most relevant. These findings suggest that EM words are related to a complex and dynamic emotion process which involves different components, culminating in the categorization (or labeling) of an emotion episode (feeling). EL words, in turn, are closely related to an appraisal process in which people assess how pleasant or unpleasant (positive or negative) the referent of a word is. This appraisal process seems to be less relevant in the definition of EM words. Overall, these findings demonstrate the importance of combining two-dimensional models with multi-componential models of emotion to provide a comprehensive understanding of the human affective space.

## Data availability statement

The datasets presented in this study can be found in online repositories. The names of the repository/repositories and access number(s) can be found at: https://osf.io/hxcm2/?view_only=74adb248fc88443fabc5ad1daa6abbc6.

## Ethics statement

This study was approved by the Ethics Committee for Research on People, Society and the Environment of the Universitat Rovira i Virgili (CEIPSA-2021-PR-0044). The studies were conducted in accordance with the local legislation and institutional requirements. The participants provided their written informed consent to participate in this study.

## Author contributions

Á-AB: Conceptualization, Methodology, Investigation, Data curation, Writing-original draft. MG: Conceptualization, Methodology, Project administration, Validation, Writing-review and editing. PF: Conceptualization, Project administration, Supervision, Funding acquisition, Writing-review and editing.

## Figures and Tables

**Figure A1:**
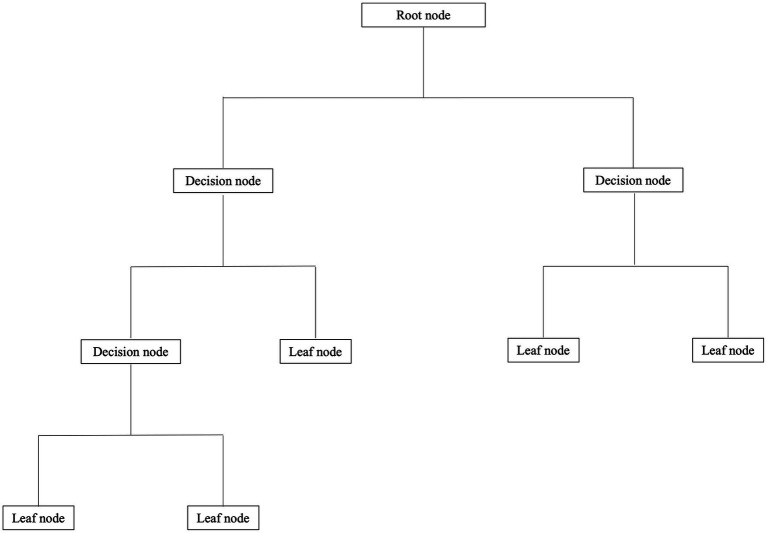
Example of a decision tree.

## Appendix

### Random forest

Breiman (2001a) developed the Random Forest (RF) algorithm as an extension of decision trees. RF is a powerful machine learning algorithm used for classification and regression problems. The RF is an ensemble method, which is a technique that typically combines the predictions of hundreds of decision trees. A decision tree is assembled based on a training dataset (Rokach and Maimon, 2005). It is a tree-like structure that divides the input data into different subsets according to the values of various features or dimensions. The decision tree structure consists of a root node, a decision node, and a leaf node (see Figure A1).

The decision tree starts with the root node, which evaluates the whole data set in terms of one feature and separates the data into those that meet the root criterion and those that do not (Rokach and Maimon, 2005). For example, the root node can divide the data into observations with a value greater or equal to 6.0 within the valence dimension. Afterward, each decision node divides the data into subgroups by testing a single feature until finding a leaf node that only contains observations representing a pure class (e.g., EM group). Therefore, the decision tree algorithm continues to create decision nodes until it separates the data into groups containing only one unique class label (pure nodes). In some cases, the decision tree algorithm cannot decompose the data into pure nodes; however, it is always possible to set various hyperparameters, such as maximum depth. The “max_depth” determines the number of decision nodes performed in the tree (Probst et al., 2019). For instance, setting a maximum depth to 2 in Figure A1 would result in a tree with three leaf nodes and one decision node. Therefore, the “max_depth” parameter sets a limit to stop the node splitting.

First, the algorithm divides the dataset into a training and a test dataset to create a random forest (Breiman, 2001a; Fife and D’Onofrio, 2022). The training set is used to train the prediction model, and the test set is used to evaluate how well the model performs with unseen data once the prediction model is created. One of the most important characteristics of RF is that each individual tree within the forest is grown using a bootstrapped sample with replacement (Archer and Kimes, 2008), which refers to randomly selecting observations from the training set. This random selection is made with replacement, meaning that an observation can be selected multiple times for the same tree, so that each tree is trained with different observations (Probst et al., 2019).

In addition to bootstrapped samples, a second important aspect of RF is that each individual tree is formed by randomly selecting, at each node, a random feature to evaluate the decision node (Breiman, 2001a; Probst et al., 2019; Fife and D’Onofrio, 2022). For example, the RF generally samples *√m* features to determine the root node, with *m* being the total number of variables that the dataset contains. After randomly sampling the features, the algorithm, among all possible splits, selects the feature that best splits the data. The random sampling of features continues at each individual node until a leaf node or “max_depth” is reached (Probst et al., 2019). One of the key benefits of random feature selection is that is helps to reduce the variance of the model. In addition, by randomly selecting a subset of features at each node, the model is less likely to be influenced by one individual feature. In general, bootstrapped sampling with replacement and random feature selection helps to create a more diverse set of decision trees. Consequently, the correlation between each individual decision tree within the forest decreases, reducing the chances of overfitting and improving the overall performance of the model.

Once all the decision trees are built, the trained model can be used to make predictions over the test data set. Based on input data (test dataset), the algorithm examines the predictions made by each tree and selects the class that the majority of trees have predicted (majority voting) (Speiser, 2021). For example, if a random forest contains 100 decision trees, 70 of which predict class EM and 30 predict class EL, then the random forest would predict class EM for that particular observation.

The RF algorithm has shown excellent results compared to other techniques, such as logistic regression, decision trees, neural nets, and k-nearest neighbors, among others (Breiman, 2001b). Another key advantage of RF is that it can detect interactions and non-linear effects without requiring the explicit modeling of these relationships (Fife and D’Onofrio, 2022). The RF can capture interactions by building multiple decision trees and averaging their predictions. Thus, exposing each feature to various combinations provides a comprehensive understanding of the complex relationship that a feature may have with the response.

RF is also capable of calculating feature importance using the Variable Importance (VI) metric. Variable importance measures the extent to which a feature contributes to the outcome or prediction of a model by calculating whether the prediction error increases or decreases when a specific feature is included in the model (Archer and Kimes, 2008; Strobl et al., 2009; Fife and D’Onofrio, 2022). It helps to select the most important features of the model in predicting an outcome. There are numerous ways to calculate the VI, such as the Gini index, z-score, permutation importance, or mean decrease in accuracy.

In addition, selecting a subset of the most relevant features might be helpful when working in high dimensional settings. The latter can be achieved by using Recursive Feature Elimination (RFE). RFE is a backward selection method that aims to reduce the number of features while preserving the predictive accuracy of the model (Wang et al., 2022). First, it removes the feature with the lowest relevance to the overall predictive performance. Subsequently, it recalculates the feature relevance and eliminates the second least relevant feature. This last process continues until only one feature remains. This approach is efficient when working with correlated features since the impact of each feature on the predictive performance may change at each step of the backward elimination (Gregorutti et al., 2017). Therefore, rather than just calculating the relevance of each feature once, recalculating it at every step improves the feature selection process.
